# Cook County Medical Society

**Published:** 1854-04

**Authors:** Hiram Smith

**Affiliations:** Secretary pro tem.


*Cook County Medical Society.*

THE regular monthly meeting of this Society was held in the office of Dr. N. S. DAVIS, on the 4th of April. The President and Secretary both being absent, the meeting was called to order by the Vice President, and Dr. H. Smith appointed Secretary pro tem. The communication of facts and cases being called for, Dr. D. A. Colton alluded to some cases of remittent fever, and made the inquiry whether, when sulphate of quinine given in two or three grain doses produced unpleasant symptoms, such as ringing in the ears, nervousness; &c., the *dose* should be *incraesed* with a view to induce the sedative effect of the remedy; or whether it should be modified by the addition of other remedies or superceded altogether?

Dr Schneider related some interesting facts in relation to the use of quinine in one of the military hospitals in Europe, from which he inferred that quinine was very often borne with less cerebral disturbance if given immediately after the operation of a mild cathartic, or in combination with a few grains of pulv. rhei. Similar facts had also been noticed by other members of the Society.

Dr. N. S. Davis related some cases of erysipelas; one particularly of the face and scalp, in which the muriated tinct. of iron was given in doses of 20 drops every four hours, with prompt and decided benefit. The tinct. of iron was not exhibited until the third or fourth day; and had been preceded by alterative doses of calomel combined with opium.

On inquiry among the members, it was found that erysipelas had been more prevalent than usual in the city, during the last two weeks. Two or three deaths had been reported from that disease; and Dr. Davis said he had met with six cases recently in his own practice, and all but one in the southern part of the city.

The reading of essays having been called for, Dr. F. Schneider, read a paper headed, "A new method of extracting the retained placenta." The paper of Dr. Schneider referred chiefly to those cases of retention of the placenta requiring the use of the proper forceps for its extraction; and the novelty proposed was in the mode of making the *tractive* force. He said the chief immediate dangers encountered in removing adherent and retained placentas, were hœmorrhage, and inversion of the fundus of the womb. The Doctor related some cases which had led him to the conclusion that if, when the retained body was seized by the forceps, the traction was preceded and accompanied by a twisting or rotary motion of the instrument, the adherent parts would be detached with less liability to hœmorrhage or inversion, while the same manipulation would strengthen the placenta itself, and render it less liable to be torn so as to leave a part behind. Dr. Schneider's paper was an interesting one and elicted some discussion.

He was also requested to place a copy on file among the papers of the Society.

The present meeting was the proper one for the annual election of officers; but several of the older members of the society being absent, that business was postponed.

And on account of the meeting of the American Medical Association on the first Tuesday in May, it was resolved to hold the next monthly meeting on the *second Tuesday* of May next; after which the Society adjourned.

HIRAM SMITH, M. D.,
*Secretary pro tem.*

